# Impact of oxygen as a vasodilator on respiration-related Gontan hemodynamics assessed by real-time phase-velocity MRI

**DOI:** 10.1186/1532-429X-17-S1-Q82

**Published:** 2015-02-03

**Authors:** Hermann Koerperich, Katja Müller, Peter Barth, Andreas Peterschröder, Jürgen Gieseke, Deniz Kececioglu, Wolfgang Burchert, Kai T Laser

**Affiliations:** 1 Institute for Radiology, Nuclear Medicine and Molecular Imaging, Heart and Diabetes Center. North-Rhine Westfalia, Bad Oeynhausen, Germany; 2 Ruhr-University of Bochum, Bochum, Germany; 3 Department of Surgery for Congenital Heart Defects, Center for Congenital Heart Defects, Bad Oeynhausen, Germany; 4 University of Bonn, Bonn, Germany

## Background

Inhalative administration of oxygen increases pulmonary blood flow by vasodilation. Currently, flow rate quantification using conventional two-dimensional phase-contrast MRI (PC-MRI) is not capable to study short-term effects due to averaging of flow information. The aim of this study was to examine the impact of oxygen supply on respiration-related flow rate variations by non-electrocardiographic triggered real-time phase-contrast MRI (PC-MRI) in patients after total cavo-pulmonary connection (Fontan).

## Methods

Real-time PC-MRI using EPI (TR/TE_eff_ /flip=12-14ms/3.3ms/40°, temporal resolution=24-28ms) was applied to study respiration-driven blood flow fluctuations in the ascending aorta (AAo), superior vena cava (SVC) and inferior vena cava (IVC) under normal breathing prior and after administration of 100% oxygen (4l/min; 10min) in 27 Fontan patients (mean age=17.2±7.5yrs; range: 6.9 to 37.9a). According to a 13 parameters risk score, patients were grouped as having good (N=19) or compromised hemodynamics. Respiration-dependent flow rates were virtually generated by dividing the respiration curve into four segments: expiration, end-expiration, inspiration and end-inspiration.

## Results

Under oxygen administration heart rate decreased from 77.6±15.7 to 75.9±14.6bpm (P=0.027). Mean body-surface indexed stroke volumes (SV_i_) increased slightly but only aortic SV_i_ change was statistically significant (pre:41.5ml/m2; post:43.6ml/m2;P=0.043). Effects were more pronounced in pts with good Fontan circulation (HR:75.2±15.9 vs. 70.8±16.4bpm,P=0.000; SV_i_(AAo):46.1±11.1 vs. 48.7±12.7ml/m2,P=0.045) than in pts with failing Fontan (HR:84.1±9.8 vs. 83.6±9.5bpm,P=n.s.;SV_i_(AAo):30.4±6.2 vs. 31.1±5.8ml/m2,P=n.s.). Mean absolute blood flow in Fontan patients for AAo, IVC and SVC was 3.14±0.78, 1.84±0.52 and 0.93±0.38L/min/m2 which remained unchanged under inhalative oxygen (3.13±0.77, 1.88±0.65 and 0.89±0.32L/min/m2).

Aortic flow rate was elevated during expiration (4.8±5.5%) and decreased during inspiration (-2.9±8.7%) in relation to mean blood flow, highest flow was detected during inspiration in IVC (81.1±55.3%) and SVC (18.6±30.8%) and lowest flow during expiration (IVC:-85.7±56.9%,P<0.05) and end-inspiration (SVC:-21.0±19.2%,P<0.05), respectively. Differences were unchanged under oxygen supply in AAo (4.4±5.8%,-3.4±8.6%,n.s.) and SVC (25.3±32.7%,-18.8±33.4%,n.s.), whereas were slightly elevated in IVC (94.5±66.3%,-98.0±69.0%). Differences were only statistically significant for end-expiratory IVC blood flow (P=0.033).

## Conclusions

Real-time PC-MRI allows quantification of respiratory-related flow rate fluctuations in Fontan patients. In this study inhalative oxygen resulted in an increase of aortic stroke volume and a decrease of heart rate only in case of good hemodynamics. As patients with compromised Fontan circulation did not show significant changes under oxygen supply this provocation may be promising for prognostic aspects and the follow up under anti pulmonary-hypertensive medication.

## Funding

Sponsored by the Fördergemeinschaft Deutsche Kinderherzzentren (project Nr.W-BDO-019/2013).

